# Temporal trends and age-period-cohort effects of tuberculosis incidence among older adults in western China, 2005–2020

**DOI:** 10.1186/s12877-026-08056-8

**Published:** 2026-08-07

**Authors:** Changping Liu, Junqi Li, Fei Huang, Jingjuan Ren, Mengdi Zhang, Yanfang Gao, He Zhu, Qiqi Wang, Ni Wang

**Affiliations:** 1 Office of Epidemiology (Technical Guidance Office for Patriotic Health Work), Chinese Center for Disease Control and Prevention (Chinese Academy of Preventive Medicine), Beijing, China; 2 Center for Tuberculosis Control and Prevention, Chinese Center for Disease Control and Prevention (Chinese Academy of Preventive Medicine), Beijing, China; 3 Chaoyang Medical Insurance Center, Beijing, China; 4 Office of Finance, Chinese Center for Disease Control and Prevention (Chinese Academy of Preventive Medicine), Beijing, China; 5 China Center for Health Department Studies, Peking University, Beijing, China

**Keywords:** Tuberculosis, Incidence, Older adults, Temporal trends, Age-period-cohort analysis, Western China

## Abstract

**Background:**

Tuberculosis remains a major public health challenge in China, and older adults in western China bear a disproportionate burden. This study examined the temporal trends in TB incidence among older adults in western China and assessed the independent effects of age, period, and cohort.

**Methods:**

TB incidence data for adults aged 60 years and above in western China from 2005 to 2020 were obtained from China’s Tuberculosis Information Management System. Joinpoint regression was used to characterize temporal trends and identify change points overall and by sex and residence, reporting annual percentage change (APC) and average annual percentage change (AAPC). An age–period–cohort model using the intrinsic estimator method was conducted to estimate the independent age, period, and birth cohort effects.

**Results:**

The age-standardized incidence rate (ASIR) decreased from 172.29 to 113.10 per 100,000, showing a non-significant declining tendency (AAPC=-2.91%, 95% CI: -5.95%, 0.22%), with higher rates in males and in rural areas. Declines were statistically significant among males (AAPC=-3.40%, 95% CI: -5.78%, -0.96%) and in urban areas (AAPC= -5.05%, 95% CI: -8.14%, -1.85%). Joinpoint analysis identified three change points, with a moderate decline during 2007–2015 (APC= -4.80%, 95% CI: -6.64%, -2.92%), a sharp decline during 2018–2020 (APC =-20.82%, 95% CI: -31.66%, -8.27%) and non-significant increases in 2005–2007 and 2015–2018. Age–period–cohort analysis showed an inverted U-shaped age effect, with the relative risk (RR) peaking at 70–74 years (RR = 1.132, 95% CI: 1.127, 1.136) and declining at 80–84 years (RR = 0.801, 95% CI: 0.796, 0.806). Period effects decreased over time, showing higher risks among males and urban residents during 2006–2010, but this pattern reversed during 2016–2020. Cohort effects generally declined, with the lowest risk in the 1956–1960 cohort (RR = 0.883, 95% CI: 0.877, 0.889).

**Conclusions:**

TB incidence among older adults in western China showed a non-significant overall downward trend from 2005 to 2020, with marked reductions after 2018. Disparities by sex and residence, together with variations in age, period and cohort effects highlight the need for targeted TB prevention and control, particularly among older males and rural residents.

## Background

Tuberculosis (TB) is a chronic infectious disease caused by *Mycobacterium tuberculosis* [1] and remains a major public health challenge worldwide and in China. The World Health Organization reported that there were approximately 10.7 million new TB cases globally in 2024, with an estimated incidence rate of 131 per 100,000 population. In China, an estimated 696,000 new TB cases were reported, corresponding to an incidence rate of 49 per 100,000 population, making China rank fourth globally in terms of TB disease burden [2].

Western China presents a severe disease burden, with a significantly higher TB incidence than that in the central and eastern regions [3, 4]. Provincial-level evidence indicates that the western provinces such as Xinjiang had a multiyear average pulmonary TB notification rate of 155 per 100,000, substantially higher than rates in central and eastern provinces [3]. This disparity has been linked to relatively low levels of economic development, uneven distribution of health resources, and complex geographical conditions in western China that constrain access to timely diagnosis and treatment [5–7]. Poverty, as another significant risk factor for TB, remains more prevalent in underdeveloped western regions than in the eastern and central areas [8]. As a result, these factors underscore the need to reduce regional disparities and prioritize western China in national TB prevention and control.

Additionally, aging poses a great challenge to TB control and prevention in China, and older adults have become a highly vulnerable population [9–11]. Age-related immune decline, chronic comorbidities, reduced tolerance to treatment, and diagnostic challenges may increase susceptibility to TB among older adults [11, 12]. The proportion of individuals aged 60 years and over in China increased from 12.4% in 2010 to 21.1% in 2023 [13] and is projected to reach approximately 28% by 2040 [14]. The proportion of individuals aged 60 years and above in newly diagnosed TB cases without HIV infection increased from 23.82% in 1990 to 37.54% in 2019 [10]. The reported incidence of TB among individuals aged 60 years and above in China has been approximately 2 to 3 times higher than that of young people [15]. Modeling studies suggest that failure to curb TB incidence among older adults, driven largely by reactivation disease in an aging population, may substantially hinder China’s progress toward incidence targets set by the End TB Strategy [16].

Previous studies have examined TB incidence in China across different age groups and regions [15]. However, evidence remains limited regarding the joint effects of age, period and birth cohort on TB incidence among older adults, especially in western China. This constrains the identification of high-risk populations and the development of targeted prevention strategies. Therefore, this study used Joinpoint regression to describe the temporal trends in TB incidence among older adults (aged ≥ 60 years) in western China from 2005 to 2020 and further assess differences in TB incidence by sex and residence. In addition, an age–period–cohort model using the intrinsic estimator (IE) method was applied to quantify the independent effects of age, period, and birth cohort on TB incidence among older adults, providing evidence to support more precise TB control strategies.

## Methods

### Data sources

This was a retrospective observational study based on a secondary analysis of routinely collected surveillance data from the Tuberculosis Information Management System (TBIMS) in China. Officially launched nationwide in January 2005, TBIMS enables TB control institutions to report individual TB case information in real time [17]. In this study, annual TB incidence for older adults aged 60 years and above in western China from 2005 to 2020 was extracted and stratified by sex (male and female), age group (60–64, 65–69, 70–74, 75–79, 80–84 and 85 + years) and residence (urban and rural areas). The inclusion criteria were reported TB cases in adults aged 60 years and above from western China during 2005–2020, including both pulmonary TB and extrapulmonary TB cases. The exclusion criteria were cases aged < 60 years, cases from non-western regions, and cases from counties or districts with unclear urban–rural classification.

Counties, including county-level cities, were classified as rural areas, while municipal districts were classified as urban areas, based on the administrative division recorded in the surveillance data. This administrative-boundary-based classification has been commonly used in Chinese epidemiological studies. According to the classification of the National Bureau of Statistics of China, China is divided into eastern, central, western and northeastern regions. Based on the Seventh National Population Census, the western region accounted for 27.12% of the total population in 2020 [18], and it includes 12 provinces, autonomous regions and municipalities: Inner Mongolia, Guangxi, Chongqing, Sichuan, Guizhou, Yunnan, Shaanxi, Gansu, Qinghai, Tibet, Ningxia, and Xinjiang. The 2010 Chinese Census population was used as the standard population to calculate the age-standardized incidence rate (ASIR), as it is widely adopted in epidemiological studies in China and facilitates comparability with domestic studies and temporal analyses.

This study was approved by the Institutional Review Board of the Chinese Center for Disease Control and Prevention (approval No. 202224).

### Statistical analysis

#### Joinpoint regression

Temporal trends in TB incidence among older adults aged 60 years and above in western China from 2005 to 2020 were evaluated using the Joinpoint regression model [19]. This method applies piecewise regression to divide the study period into several segments connected by joinpoints, allowing the identification of statistically significant changes in trends over time. Regression models were fitted to the natural logarithm of the TB incidence rate. The annual percentage change (APC) was calculated for each segment; the average annual percentage change (AAPC) was calculated to summarize the overall trend across the entire study period. APC and AAPC were considered statistically significant when compared to 0 with *p-values* of < 0.05. In this study, crude incidence rates (CIRs) and ASIRs were analyzed to estimate AAPC overall and by sex (male and female) and residence (urban and rural). CIRs were presented only as descriptive indicators of the original incidence level, while ASIR-based models were further analyzed to identify joinpoints and estimate segment-specific APCs. In addition, for age-specific analyses, AAPC values were estimated to summarize the overall temporal trend in each age group.

In the Joinpoint analysis, the software’s built-in “Standard Error (Calculated)” option was adopted to address heteroscedasticity, and an uncorrelated error model was applied. All analyses were conducted using Joinpoint (version 5.0.2; National Cancer Institute, Calverton, MD, USA).

#### Age–period–cohort analysis

An age–period–cohort model was used to examine the independent effects of age, period, and birth cohort on TB incidence among older adults. The analysis has been widely used in epidemiological and demographic research to investigate long-term temporal patterns of disease occurrence [20]. However, due to the linear correlation among age, period, and cohort (cohort=period-age), it is difficult for conventional age–period–cohort models to eliminate the interaction [21], known as a non-identification problem [22]. To address this issue, the intrinsic estimator (IE) method was proposed [23] and has been extensively applied in population health studies. In this study, “age” was defined as the biological age, and the age effect referred to the effects of different age groups on TB incidence. “Period” referred to the calendar year in which TB cases were diagnosed, and the period effect referred to the influences of temporal changes such as advancements in medical technology and diagnostic methods. “Birth cohort” was defined according to year of birth, and the cohort effect referred to the differences attributable to early-life exposures, such as socioeconomic conditions, environmental factors and lifestyle that may influence TB incidence over the life course [15, 24].

In the model with the IE method, equal intervals for age, period, and cohort were required. Because the age variable in the surveillance system was already grouped into 5-year categories, a 5-year interval was adopted consistently for all three dimensions. Accordingly, TB incidence data were categorized into five age groups (60–64, 65–69, 70–74, 75–79, and 80–84 years), three calendar periods (2006–2010, 2011–2015, and 2016–2020), and seven birth cohorts (1926–1930, 1931–1935, 1936–1940, 1941–1945, 1946–1950, 1951–1955, and 1956–1960). Data for the open-ended 85 + age group or year 2005 were excluded from the age–period–cohort analysis because they could not be incorporated into complete 5-year age and period groupings required by the model. Since adjacent birth cohorts partially overlap, the middle year of the corresponding cohort range was often used to represent a 5-year birth cohort [15]. Accordingly, individuals aged 75–79 and 80–84 years from 2006 to 2010 corresponded to birth-year ranges from 1927 to 1935 and 1922 to 1930, respectively, and they were therefore assigned to the 1931–1935 and 1926–1930 birth cohorts. The age–period–cohort model expression could be written as [21]

$$\begin{aligned} \:\mathrm{Y}&=\mathrm{log}\left(\mathrm{M}\right)={\upmu\:}+{\upalpha}{\mathrm{a}\mathrm{g}\mathrm{e}}_{1}\\&+{\upbeta}{\mathrm{p}\mathrm{e}\mathrm{r}\mathrm{i}\mathrm{o}\mathrm{d}}_{1}+{\upgamma}{\mathrm{c}\mathrm{o}\mathrm{h}\mathrm{o}\mathrm{r}\mathrm{t}}_{1}+{\upepsilon\:} \end{aligned}$$

where M stands for the TB incidence of different age groups; α, β, and γ stand for the regression coefficients of age, period and cohort effects of the model; $\:{\upmu\:}$ and $\:{\upepsilon\:}$ represent the intercept item and the random error. In the age-period-cohort analysis using the IE method, model parameters (α, β, γ) were converted to exponential values, indicating the relative risk (RR) of incidence for a particular age, period, and birth cohort relative to each average level. The age–period–cohort analysis was conducted in Stata 18.0 (StataCorp, College Station, TX, USA) using the apc_ie procedure to estimate model parameters.

## Results

### Joinpoint regression analysis of TB incidence

**Fig. 1:**
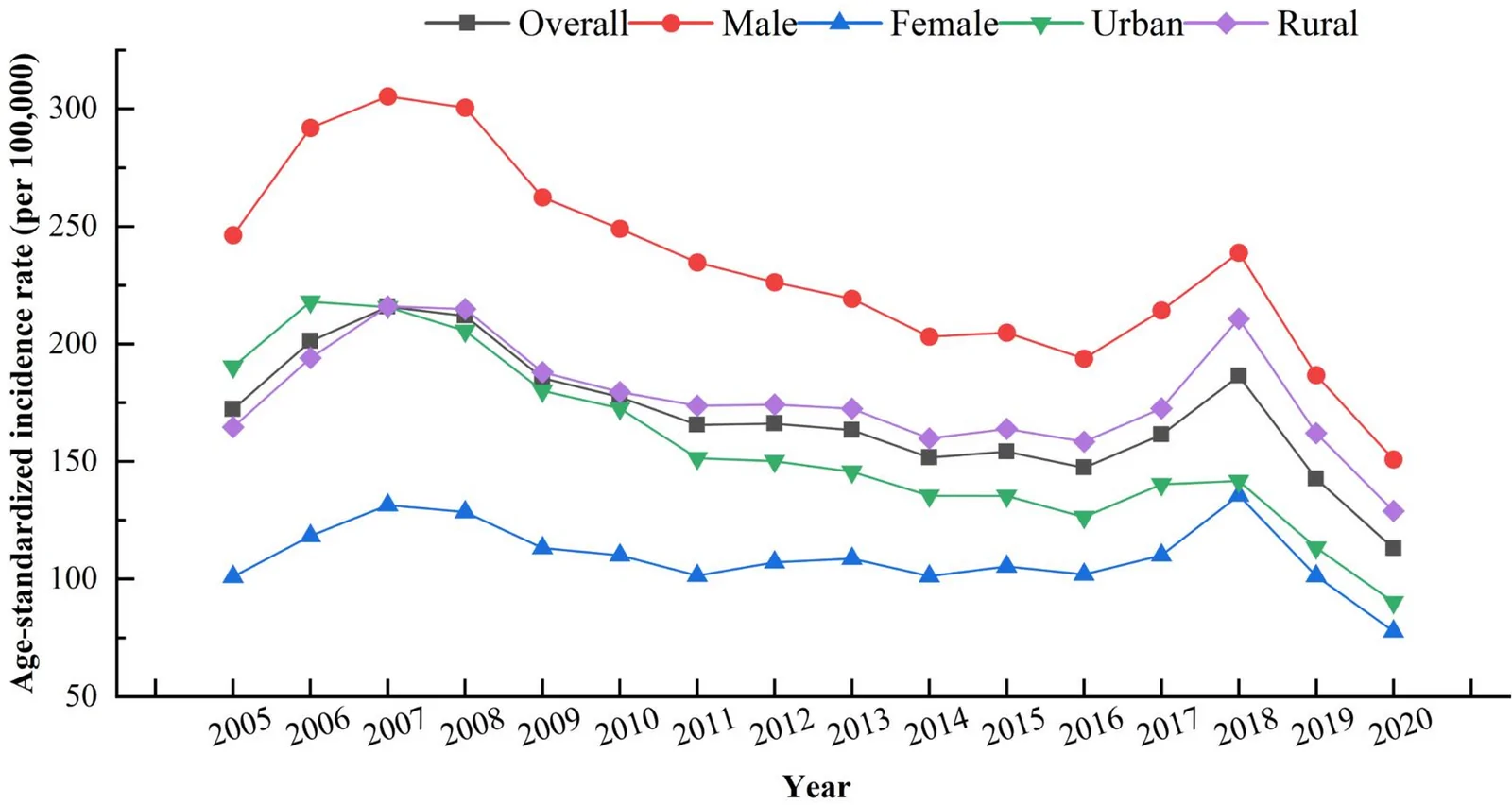
Temporal trends in age-standardized TB incidence among people aged ≥ 60 years in western China, 2005–2020

From 2005 to 2020, a total of 1,344,478 TB cases were reported among older adults aged 60 years and above in western China. As shown in Fig. 1 and Table 1, from 2005 to 2020, the ASIR for TB among older adults aged 60 years and above in western China declined from 172.29 to 113.10 per 100,000, although this overall decrease did not reach statistical significance (AAPC=-2.91%, 95% CI: -5.95%, 0.22%). Similar non-significant decreasing tendencies were observed in females and rural areas. Statistically significant decreases were shown in males (AAPC=-3.40%, 95% CI: -5.78%, -0.96%) and in urban areas (AAPC=-5.05%, 95% CI: -8.14%, -1.85%). In 2020, the ASIR was higher in males (150.98 per 100,000) than in females (77.82 per 100,000), and in rural areas (129.02 per 100,000) than in urban areas (90.19 per 100,000).

**Table 1:** TB incidence among older adults in western China by sex and residence (per 100,000), 2005–2020

Year	Male		Female		Urban		Rural		Overall	
	CIR	ASIR	CIR	ASIR	CIR	ASIR	CIR	ASIR	CIR	ASIR
2005	249.47	246.36	103.51	101.08	192.98	190.45	168.88	164.69	176.08	172.29
2006	292.97	292.07	120.68	118.42	220.04	218.08	197.48	194.15	204.22	201.20
2007	304.74	305.45	133.94	131.54	216.76	215.78	219.22	216.10	218.44	215.92
2008	299.94	300.64	130.55	128.53	206.23	205.64	217.75	214.95	214.11	211.97
2009	262.30	262.47	115.00	113.28	180.95	180.14	190.51	188.05	187.48	185.55
2010	248.52	249.08	111.58	110.19	173.11	172.69	181.63	179.64	178.93	177.45
2011	234.19	234.79	102.68	101.49	151.54	151.49	174.73	173.83	166.88	165.64
2012	228.01	226.33	107.57	107.18	150.97	150.20	175.66	174.33	167.28	166.15
2013	220.92	219.34	109.09	108.72	146.49	145.74	173.75	172.50	164.52	163.45
2014	204.55	203.21	101.22	101.29	136.14	135.53	160.43	159.92	152.35	151.75
2015	206.06	204.87	105.47	105.44	135.96	135.42	164.51	163.94	154.94	154.31
2016	194.78	193.84	101.98	102.05	126.47	126.43	158.86	158.46	147.79	147.44
2017	215.80	214.38	110.49	110.19	140.93	140.40	173.56	172.67	162.30	161.44
2018	240.23	238.88	136.38	135.64	142.38	141.82	212.14	210.80	187.59	186.44
2019	184.75	186.82	100.54	101.39	112.10	113.40	161.05	162.13	141.57	142.77
2020	150.33	150.98	77.77	77.82	89.95	90.19	128.85	129.02	112.88	113.10
AAPC (%)	-3.54	-3.40	-2.02	-1.83	-5.18	-5.05	-1.89	-1.70	-3.09	-2.91
Test Statistic	-2.655	-2.716	-0.983	-0.874	-3.043	-3.067	-1.097	-1.002	-1.922	-1.822
P	0.008	0.007	0.326	0.382	0.002	0.002	0.273	0.316	0.055	0.068

For each subgroup, the rows labeled Test Statistic and P refer to the statistical test of whether the AAPC is significantly different from zero. In the Joinpoint software, a two-sided t-test is used for models with 0 joinpoints, whereas a two-sided z-test is used for models with 1 or more joinpoints

*CIR* crude incidence rate, *ASIR* age-standardized incidence rate, *AAPC* average annual percentage change

As shown in Table 2, Joinpoint regression analysis identified three change points in the temporal trend of TB incidence among older adults in western China from 2005 to 2020. Significant declining trends were observed during periods 2007–2015 and 2018–2020, with APCs of -4.80% (95% CI: -6.64%, -2.92%) and − 20.82% (95% CI: -31.66%, -8.27%), respectively. Two non-significant increases were observed in 2005–2007 and 2015–2018. In the sex and residence specific analyses, three joinpoints were also identified, and the most pronounced decline was consistently observed during 2018–2020 across all subgroups. Table 3 summarizes the overall age-specific variations in TB incidence from 2005 to 2020 using AAPC values. TB incidence among older adults in western China varied across age groups, with differing magnitudes of change. Statistically significant declines were observed in the 60–64-year age group (AAPC = − 4.71%) and the 70–74-year age group (AAPC = − 3.23%).

**Table 2:** Joinpoint regression results for age-standardized TB incidence among older adults in western China by sex and residence, 2005–2020

Type	Joinpoints	Year	APC (95% CI) (%)	AAPC (95% CI) (%)
Overall	3	2005–2007	10.06 (-5.79, 28.57)	-2.91(-5.95, 0.22)
		2007–2015	-4.80* (-6.64, -2.92)	
		2015–2018	7.81 (-6.51, 24.32)	
		2018–2020	-20.82* (-31.66, -8.27)	
Sex				
Male	3	2005–2007	9.95 (-2.51, 24.00)	-3.40*(-5.78, -0.96)
		2007–2015	-5.68* (-7.11, -4.23)	
		2015–2018	6.69 (-4.64, 19.37)	
		2018–2020	-19.54* (-28.45, -9.53)	
Female	3	2005–2007	11.06 (-10.31, 37.52)	-1.83 (-5.83, 2.33)
		2007–2015	-3.30* (-5.79, -0.75)	
		2015–2018	10.10 (-8.34, 32.36)	
		2018–2020	-22.41* (-35.58, -6.55)	
Residence				
Urban	3	2005–2007	4.65 (-12.90, 25.73)	-5.05* (-8.14, -1.85)
		2007–2014	-6.89* (-9.64, -4.06)	
		2014–2018	1.78 (-6.82, 11.18)	
		2018–2020	-19.70* (-32.50, -4.47)	
Rural	3	2005–2007	13.06 (-4.19, 33.42)	-1.70 (-4.94, 1.65)
		2007–2015	-4.10* (-6.05, -2.11)	
		2015–2018	9.61 (-5.55, 27.20)	
		2018–2020	-19.87* (-31.50, -6.26)	

*AAPC* average annual percentage change, *APC* annual percentage change

*P<0.05

**Table 3:** Age-specific TB incidence among older adults in western China, 2005–2020 (per 100,000)

Year	60–64	65–69	70–74	75–79	80–84	85+
2005	168.75	190.41	203.59	164.14	112.73	96.13
2006	192.14	206.74	251.17	194.32	151.43	133.60
2007	203.26	217.34	261.96	220.02	176.73	162.51
2008	197.46	211.53	255.67	224.59	172.41	166.84
2009	174.52	191.21	215.00	197.38	143.93	148.44
2010	169.21	176.37	209.60	183.38	146.25	143.84
2011	168.92	161.01	184.56	169.26	131.79	131.44
2012	160.63	174.20	193.84	175.99	132.27	73.91
2013	160.52	172.08	186.86	167.73	132.80	78.43
2014	149.42	163.11	172.69	156.70	113.18	69.91
2015	147.76	170.65	172.57	161.20	117.32	80.84
2016	151.41	159.32	155.09	149.30	109.55	80.18
2017	153.69	183.43	173.69	168.57	123.71	93.31
2018	175.76	207.22	198.06	199.64	151.29	126.49
2019	109.32	150.59	162.15	195.08	138.86	117.44
2020	87.86	114.51	131.94	149.75	127.33	78.70
AAPC (%)	-4.71	-4.04	-3.23	-0.28	0.63	-1.37
Test Statistic	-3.445	-1.794	-5.548	-0.148	0.282	-0.342
P	0.001	0.073	< 0.001	0.883	0.778	0.732

For each subgroup, the rows labeled Test Statistic and P refer to the statistical test of whether the AAPC is significantly different from zero. In the Joinpoint software, a two-sided t-test is used for models with 0 joinpoints, whereas a two-sided z-test is used for models with 1 or more joinpoints

*AAPC* average annual percentage change

### The variation in TB incidence by age, period and birth cohort among older adults in western China

**Fig. 2:**
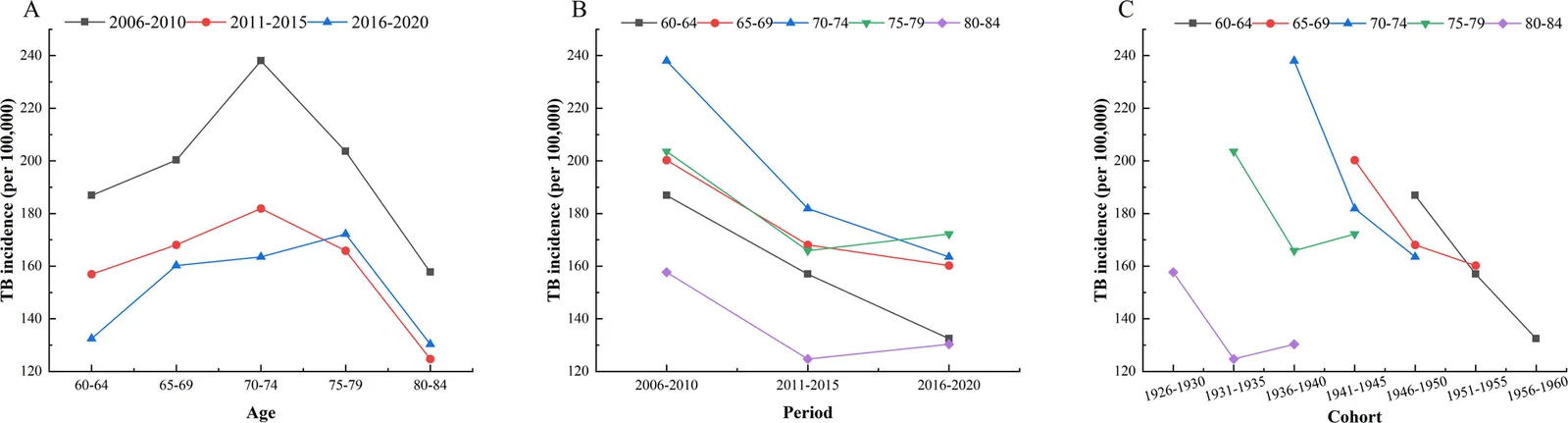
Variations in TB incidence among older adults in western China, 2006‒2020, by (**A**) Age, (**B**) Period, and (**C**) Cohort

Figure 2 illustrates the changes in TB incidence among older adults in western China by age, period, and birth cohort from 2006 to 2020. As shown in Fig. 2A, TB incidence increased with advancing age across three periods, peaking in the 70–74 or 75–79 age groups, with a lower incidence observed in the 80–84 age group. Figure 2B shows that TB incidence generally declined from 2006 to 2020 in most age groups. However, for the 75–79 and 80–84 age groups, TB incidence reached its lowest level in the 2011–2015 period and subsequently increased in the 2016–2020 period. As shown in Fig. 2C, TB incidence varied across birth cohorts. Earlier birth cohorts showed higher levels of incidence than later cohorts in most age groups, apart from the 75–79 and 80–84 age groups. A more pronounced downward trend was observed among individuals born after the 1940s.

### The age-period-cohort model analysis of TB incidence

#### Age effect

**Fig. 3:**
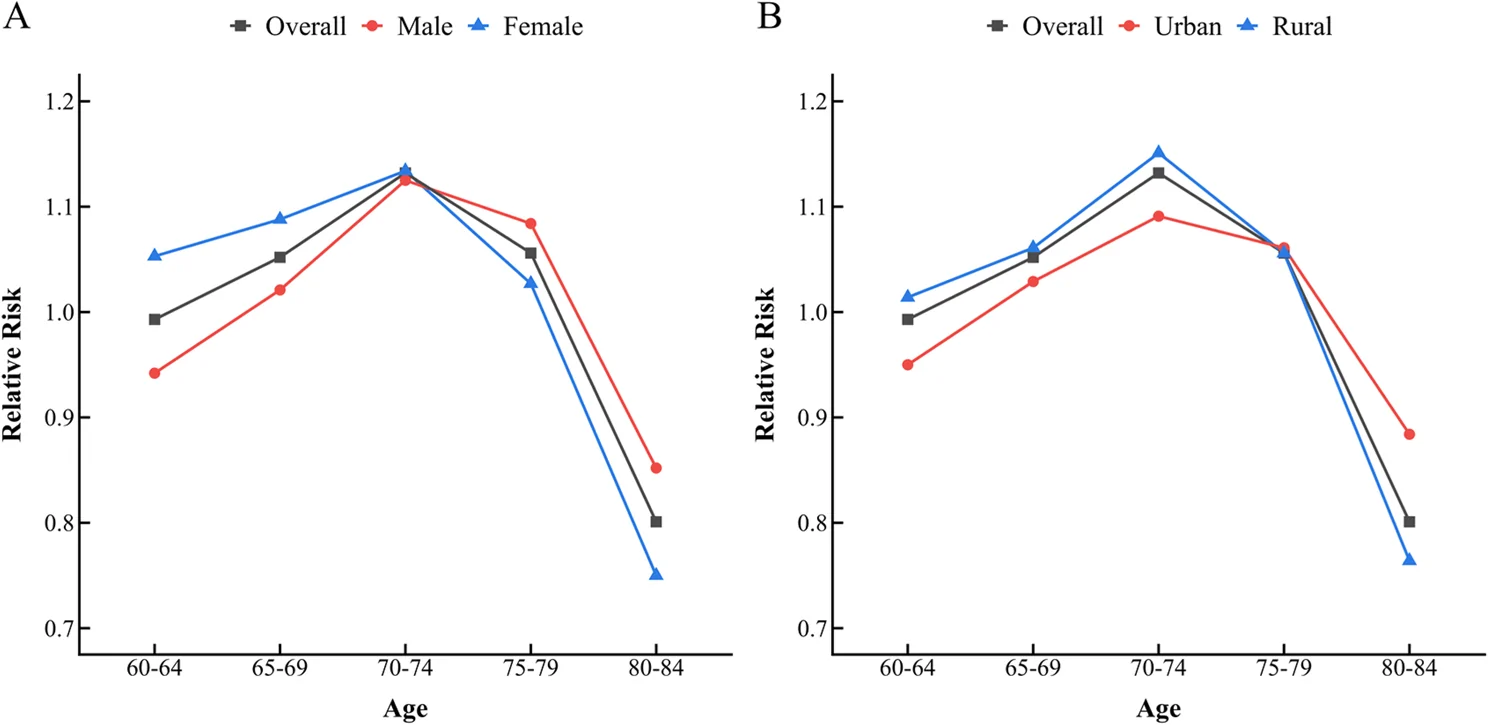
Age effects on TB incidence by (**A**) Sex and (**B**) Urban-rural residence

After adjusting for period and cohort effects, the age effect on TB incidence among older adults in western China is shown in Fig. 3. Overall, the relative risk (RR) of TB incidence increased with age from the 60–64 age group to the peak in the 70–74 age group (RR = 1.132, 95% CI: 1.127–1.136), and then decreased, reaching the lowest level in the 80–84 age group (RR = 0.801, 95% CI: 0.796–0.806). Similar patterns were observed in the age effects stratified by sex and urban-rural residence. When stratified by sex, females under 74 years showed slightly higher relative risk than males, while males had higher risks in the 75–79 and 80–84 age groups. When stratified by residence, the risk was higher in rural areas than in urban areas for the younger age groups, while urban areas showed higher risks in the oldest age group (80–84 years).

#### Period effect

**Fig. 4:**
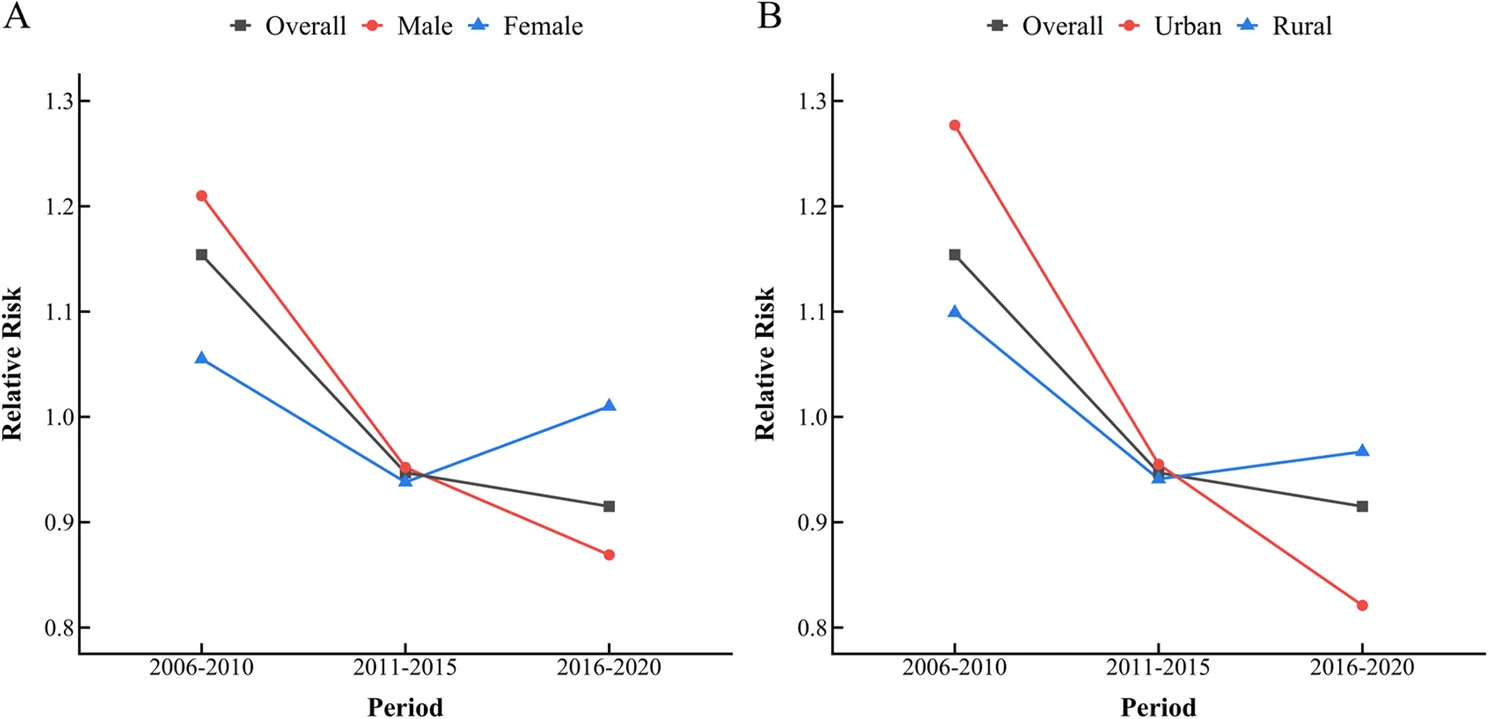
Period effect on TB incidence by (**A**) Sex and (**B**) Urban-rural residence

Figure 4 shows a decreasing period effect on TB incidence among older adults in western China after adjustment for age and cohort effects. Compared with 2006–2010 (RR = 1.155, 95% CI: 1.151–1.158), the RR decreased during 2011–2015 (RR = 0.947, 95% CI: 0.944–0.949) and remained at a lower level in 2016–2020 (RR = 0.915, 95% CI: 0.912–0.918). As shown in Fig. 4A, males (RR = 1.210, 95% CI: 1.205–1.214) had a higher risk than females (RR = 1.055, 95% CI: 1.050–1.060) during 2006–2010, whereas the risk was higher among females (RR = 1.010, 95% CI: 1.005–1.016) than males (RR = 0.869, 95% CI: 0.866–0.872) in 2016–2020. As shown in Fig. 4B, the period effect was higher in urban areas (RR = 1.277, 95% CI: 1.270–1.283) than in rural areas (RR = 1.099, 95% CI: 1.095–1.102) during 2006–2010. However, the decline in RR was more pronounced in urban areas across subsequent periods, resulting in a relatively higher risk in rural areas (RR = 0.967, 95% CI: 0.964–0.971) than in urban areas in 2016–2020 (RR = 0.821, 95% CI:0.816–0.825).

#### Cohort effect

**Fig. 5:**
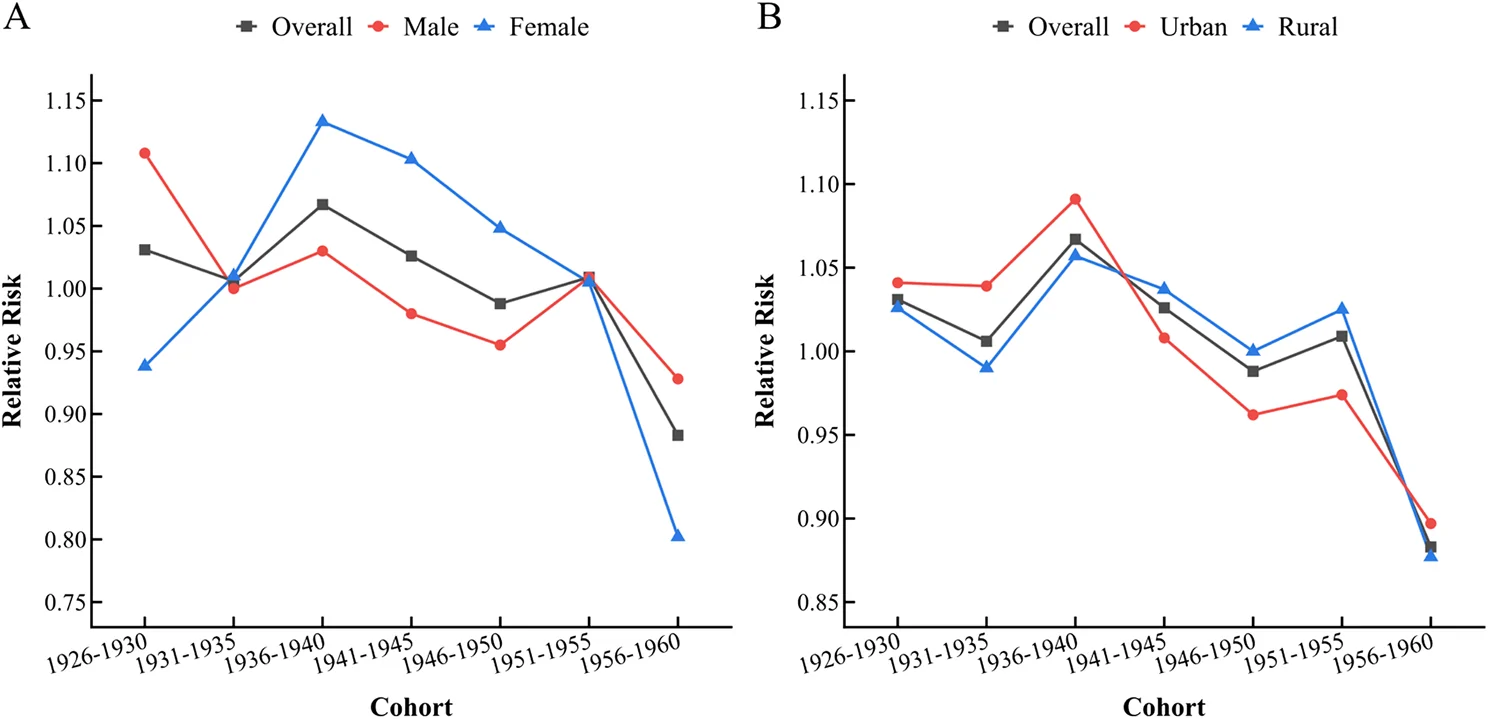
Cohort effects on TB incidence by (**A**) Sex and (**B**) Urban-rural residence

The RR for the cohort effect on TB incidence is presented in Fig. 5, indicating a general downward trend across different cohorts, with the lowest risk observed in older adults born during 1956 to 1960 (RR = 0.883, 95% CI: 0.877–0.889). The risk among females peaked in the 1936–1940 cohort (RR = 1.133, 95% CI: 1.122–1.145) and then declined steadily in later cohorts, reaching the lowest level in those born in the 1956–1960 cohort (RR = 0.802, 95% CI: 0.793–0.811). In contrast, the cohort-related variation among males was relatively modest. Cohort patterns in urban and rural areas were broadly similar to the overall trend. The RR of cohort effects was slightly higher in urban areas than in rural areas in earlier cohorts, while the decline across cohorts appeared more pronounced in urban areas.

## Discussion

This study showed a numerical decrease in TB incidence among older adults aged 60 years and above in western China from 2005 to 2020. Joinpoint regression analysis identified multiple change points in the temporal trend, with the most pronounced decline occurring from 2018 to 2020, particularly among males and in urban areas. The age–period–cohort analysis further revealed clear independent age, period, and birth cohort effects on TB incidence.

The overall downward tendency in TB incidence among older adults in western China from 2005 to 2020 is consistent with previous studies globally [9], in China [25, 26] and in eastern China [27]. The enhancement of education, economic development, medical interventions, and living conditions may have contributed to the observed decline in TB incidence among older adults [9]. Additionally, the continuous strengthening of China’s national TB control policies, including the nationwide coverage of directly observed treatment short-course strategies since 2004 [28], has likely played an important role in reducing TB transmission and improving treatment outcomes. Since 2012, China has implemented an “integrated TB control model” that involves close collaboration among the centers for disease control and prevention, designated TB hospitals and primary healthcare sectors, which may have strengthened TB case management and supported the long-term decline in TB incidence [29]. The significant decline observed during 2007–2015 may reflect the combined effects of these strengthened TB control strategies and improvements in social and health-system conditions during this period.

A modest rebound in 2015–2018, followed by the most pronounced decline during 2018–2020, may reflect both the effects of strengthened TB prevention and treatment measures and the substantial disruption of TB services during the COVID-19 epidemic in 2020. The 13th Five-Year National Tuberculosis Control Plan in 2017 emphasized improvement of the TB service system, strengthened case detection in key populations, including older adults aged 65 years and above, and full-process treatment management [30]. Several western provinces implemented related measures. For example, as one of the provinces with severe TB disease burden, Xinjiang implemented innovative interventions, which included incorporation of TB screening into routine health examinations, full-course hospitalization of active TB patients, or hospitalization during the infectious period combined with community-based “centralized medication + nutritious breakfast” [31]. Previous studies have shown that this unique model has long-term effects on reducing the reported incidence of TB after a short-term increase [32, 33], contributing to TB control in high-burden areas. In addition, other western provinces also strengthened TB control during this period, such as active TB screening for older adults in rural Sichuan [34], and the introduction of more rapid decentralized screening pathways for rifampicin-resistant TB in Yunnan [35]. Intensified TB screening and case detection in high-risk populations may temporarily increase reported TB incidence by identifying previously missed or prevalent cases, followed by a decline as transmission is curbed and case management is strengthened.

Meanwhile, the marked decline in 2020 may also have been influenced by the COVID-19 epidemic. At the national level, the COVID-19 epidemic mainly affected TB notification, follow-up examinations, and patients’ healthcare-seeking behavior [36]. For example, Ningxia Hui Autonomous Region reported delays in care during the COVID-19 period [37]. A national survey showed that about half of TB patients did not revisit hospitals for follow-up, with traffic restrictions, personal reasons, and TB service disruptions being major reasons for diagnostic delay [38]. In addition, an interrupted time-series study based on national monthly reported data from 2015 to 2023 found a significant immediate decrease in reported TB incidence at the start of the COVID-19 pandemic and a downward trend thereafter [39]. Therefore, the decline observed during 2018–2020 should be interpreted with caution, and further studies are needed to assess the impact of COVID-19-related disruptions on this trend.

The study indicated that the decrease in TB incidence was greater among older males than females in western China, although men consistently exhibited higher incidence levels throughout the study period, which was also consistent with previous studies [5, 10]. The sex disparity can be attributed to greater historical exposure to *Mycobacterium tuberculosis* among males [40]. Prolonged time spent in high-transmission places, such as workplaces and public transport [41], excessive smoking and alcohol consumption [42] and more occupational hazards [43] may increase the likelihood of TB disease. Recent evidence suggests that males have a higher risk of *Mycobacterium tuberculosis* immunoreactivity, indicating that sex differences in TB burden may originate at the stage of infection [44].

In addition, although TB incidence in rural areas was generally higher than that in urban areas during the study period, the decline was statistically significant in urban areas but not in rural areas. This pattern reflects persistent disparities in healthcare access and resource allocation between urban and rural areas. Urban areas generally benefit from better medical infrastructure, greater availability of trained healthcare workers, and more timely diagnosis and treatment of TB, which may accelerate the decrease of TB incidence. In contrast, rural areas, particularly in western China, often face longer diagnostic delays. Poverty, lower education level and poor knowledge of TB among older adults, limited health resources and weaker primary healthcare capacity in health facilities in rural areas further aggravate the diagnostic delays [9, 45]. Furthermore, compared to urban residents, rural residents are prone to have a less healthy lifestyle involving smoking and drinking [46], which is associated with TB disease.

However, given that the urban–rural classification was based on administrative divisions, rapid urbanization and administrative boundary adjustments during the study period may have introduced misclassification and affected the estimated magnitude of the urban–rural disparity. Population mobility may also have influenced the observed urban-rural disparity. Migration from less developed to more developed areas, including rural-to-urban movement, may affect where TB cases are detected and reported. Moreover, older adults are becoming an increasingly important subgroup within the migrant TB population [47].

This study adopted an age-period-cohort model with the IE method to address the non-identification problem and estimate the independent age, period and cohort effects on TB incidence. The overall age effect increased with age, peaked in the 70–74 age group, and then declined, which was consistent with previous studies globally [48]. Regarding the sex differences in age effect pattern, a slightly higher risk was found among females under 75 years and males above 75 years. The increasing risk observed from 60 to 74 years may be due to age-related immunosenescence and the high prevalence of comorbidities, such as diabetes and chronic obstructive pulmonary disease (COPD) [49]. In addition, older adults are more likely to experience reactivation of latent TB infection, which increases susceptibility to TB disease [16]. The subsequent decline in RR observed after the 70–74 age group may be explained by underdiagnosis of TB in the oldest due to atypical clinical symptoms and diagnostic challenges that are common at very advanced ages [50]. Another possible explanation is the healthy survivor effect, as individuals who survive to the oldest age groups tend to be healthier with fewer underlying risk factors, which could lower the observed risk of TB compared with those at younger old age groups [48, 51].

The period effect demonstrated a decline over time in TB risk among older adults in western China, in line with the Joinpoint regression results and previous studies [15, 26]. The decline may be attributed to sustained strengthening of TB control policies and public health interventions in China over the past two decades, especially in western China [31]. In 2016, TB screening for adults aged 65 years and above was incorporated into China’s Basic Public Health Service program, with free chest radiography provided by primary healthcare institutions [52], which may have contributed to subsequent reductions in TB risk. The decreasing RR over time was more pronounced among males than females and in urban areas than in rural areas, consistent with previous findings [15, 53]. The urban-rural difference in TB incidence was contrary to that in TB mortality, which decreased more significantly in rural areas than in urban areas among older adults [24]. This pattern may reflect differences in the effectiveness of TB control measures across population subgroups.

The cohort effect on TB incidence fluctuated and decreased markedly from the 1951–1955 birth cohort to the 1956–1960 birth cohort. The elevated risk observed in the 1936–1940 cohort may be associated with the periods of war, which could potentially exacerbate the TB burden by disrupting healthcare systems and living conditions and directly threatening population health and survival [21]. The second peak was observed in the 1951–1955 cohort, followed by a pronounced decline. This subsequent decline may be associated with improvements in living conditions, public health services, and systematic TB prevention and control after the founding of the People’s Republic of China in 1949. However, these historical interpretations should be viewed with caution, as the cohort patterns observed here may reflect population-level associations rather than direct causal effects at the individual level. Sex-stratified results indicated a higher risk of TB among females, which may be due to unequal access to healthcare, gendered socioeconomic disadvantage, and adverse early-life conditions, especially malnutrition, experienced by females during early birth cohorts. Studies have demonstrated that malnutrition was an important attributable risk factor for TB [54], and early life circumstances have been linked to health outcomes in old age [55]. Prenatal and early-life exposure to malnutrition may further increase the risk of TB in the exposed generation and their offspring [56].

This study has several limitations that should be acknowledged. Firstly, the analysis was based on routinely reported surveillance data from the Tuberculosis Information Management System. Although TBIMS has achieved nationwide coverage and improved data quality over time, underreporting of TB cases may still exist, particularly among the oldest adults. This may have led to an underestimation of TB incidence. In addition, the administrative-division-based urban–rural classification in the system may not have fully captured the actual level of urbanization, which could have affected the urban–rural comparison. Secondly, ASIRs were calculated using the 2010 Chinese Census population as the standard population. Although this choice facilitates comparison with domestic studies, it may limit direct comparability with studies using other standard populations. Thirdly, this study focused on differences by sex and urban-rural residence, but other potentially relevant factors, such as comorbidities, latent tuberculosis infection status, behavioral risk factors, and socioeconomic status, were not considered. This may limit further exploration of underlying mechanisms of TB incidence among older adults and the ability to account for additional heterogeneity or confounding. Future studies using individual-level data are needed to better clarify the pathways driving TB incidence. Finally, to satisfy the equal-interval requirement of the IE-based age-period-cohort model, age, period, and cohort were grouped using 5-year intervals, which resulted in only three period groups and required exclusion of the 85 + age group and the year 2005 from the age-period-cohort analysis. This limited the ability to detect more complex or nonlinear period patterns, constrained the interpretation of cohort effects, and may have affected the interpretation of age effects at the oldest ages as well as the representation of the earliest period and cohort boundaries. Therefore, the age-period-cohort results should be interpreted mainly as reflecting broad temporal patterns.

## Conclusions

In conclusion, this study demonstrated a non-significant declining tendency in TB incidence among older adults aged 60 years and above in western China from 2005 to 2020, with the most pronounced decline shown after 2018. The reduction varied among different groups stratified by sex and residence. The age effect showed an inverted U-shaped pattern, with risk increasing first and then decreasing with age. The period effect showed a downward trend, while the cohort effect varied across birth cohorts and decreased in risk among later birth cohorts. These findings highlight the combined influence of population aging, public health interventions, and long-term improvements in living conditions. These findings underscore the importance of strengthening TB prevention and control strategies tailored to older adults, particularly among high-risk subgroups such as males and rural residents in western China. Continued efforts to improve early detection, access to healthcare, diagnosis, treatment and management are essential to further reduce TB burden, and support China’s progress toward the End TB Strategy targets in the context of rapid population aging.

## Data Availability

The datasets analyzed during this study are available from the corresponding authors on reasonable request.

## Abbreviations

APC: Annual percentage change
AAPC: Average annual percentage change
ASIR: Age-standardized incidence rate
CI: Confidence interval
CIR: Crude incidence rate
IE: Intrinsic estimator
RR: Relative risk
TB: Tuberculosis
TBIMS: Tuberculosis Information Management System
